# A case report of *de novo* hepatocellular carcinoma after living donor liver transplantation

**DOI:** 10.1186/1477-7819-11-176

**Published:** 2013-08-06

**Authors:** Songfeng Yu, Hua Guo, Li Zhuang, Jun Yu, Sheng Yan, Min Zhang, Weilin Wang, Shusen Zheng

**Affiliations:** 1 Division of Hepatobiliary and Pancreatic Surgery, Department of Surgery, First Affiliated Hospital, School of Medicine, Zhejiang University, Hangzhou 310003, China; 2 Key Laboratory of Combined Multi-Organ Transplantation, Ministry of Public Health, First Affiliated Hospital, School of Medicine, Zhejiang University, Hangzhou 310003, China; 3 Key Laboratory of Organ Transplantation, Zhejiang Province, Hangzhou 310003, China

**Keywords:** *De novo* hepatocellular carcinoma, Liver transplantation, Living donor liver transplantation, Hepatitis B recurrence, YMDD mutation

## Abstract

Post-transplant malignancy is the major cause of later death of recipients after liver transplantation. Tumor recurrence after liver transplantation for patients with hepatocellular carcinoma in the end stage of cirrhosis has been frequently encountered. However, *de novo* hepatocellular carcinoma originating from the liver allograft has only rarely been reported. Here we reported a case of *de novo* hepatocellular carcinoma developed 2 years after living donor liver transplantation for hepatitis B-related liver cirrhosis with viral YMDD mutation. To the best of our knowledge, this is the first report of *de novo* hepatocellular carcinoma in a liver graft with recurrent hepatitis B virus infection after liver transplantation for hepatitis B-related liver cirrhosis with YMDD mutation. Moreover, the *de novo* cancer first presented as a lung mass with minimal liver involvement and was obscured by a pulmonary fungal infection.

## Background

Liver transplantation (LTx) is the sole curative strategy option for end-stage liver diseases. Transplant recipients are thought to have increased risk of developing malignancies due to their lifelong immunosuppressant requirement. This has become a major cause of late mortality after LTx [1]. Carcinomas occurring in transplant recipients after LTx have been increasingly reported. Tumor recurrence after LTx for hepatocellular carcinoma (HCC) in end-stage liver cirrhosis is frequently encountered, especially with advanced HCC patients [2]. However, *de novo* HCC originating from liver allograft in a patient who received LTx for benign disease has only rarely been reported. To date, there have only been 11 cases of *de novo* HCC after LTx, reported in 8 papers in the literature [3-11]. In this paper, we report an additional case of *de novo* HCC developed after liver graft in the setting of hepatitis B (HBV) recurrence after living donor liver transplantation (LDLT) for HBV-related end-stage liver cirrhosis with viral YMDD mutation. To the best of our knowledge, this is the first report of *de novo* HCC in a liver graft with HBV recurrence after LTx for HBV-related liver cirrhosis with YMDD mutation. Moreover, the *de novo* tumor first presented as a lung mass with minimal liver involvement, and was obscured by a pulmonary fungal infection.

## Case presentation

**Figure 1:**
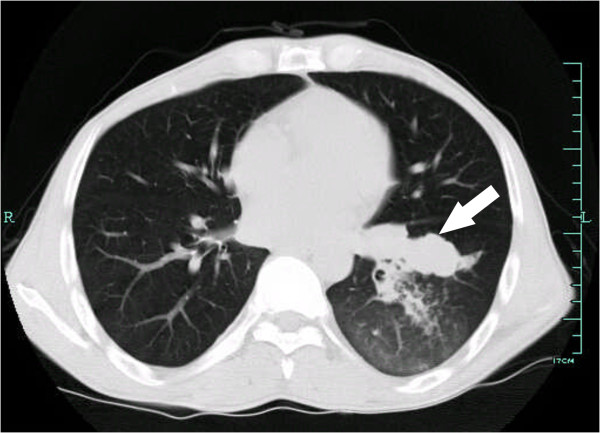
**Thoracic computed tomography 2 years after living donor liver transplantation.** The white arrow shows a nodular in the hilum of the left lung with the patchy shadow indicating pulmonary infection.

**Figure 2:**
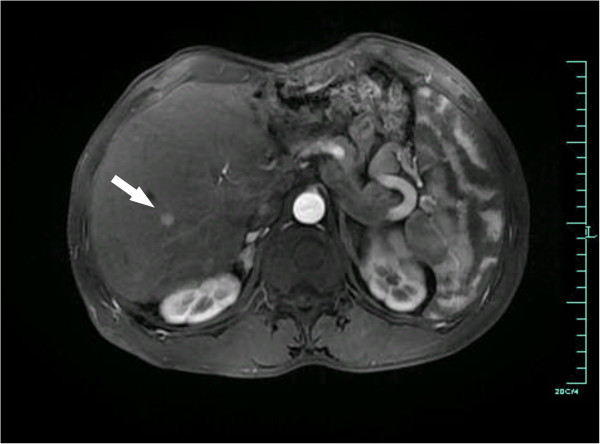
**Artery phase of enhanced abdominal MRI 2 years after living donor liver transplantation.** The white arrow indicates a 0.7-cm nodule of hepatocellular carcinoma (HCC) with arterial enhancement in segment VI of the allograft.

**Figure 3:**
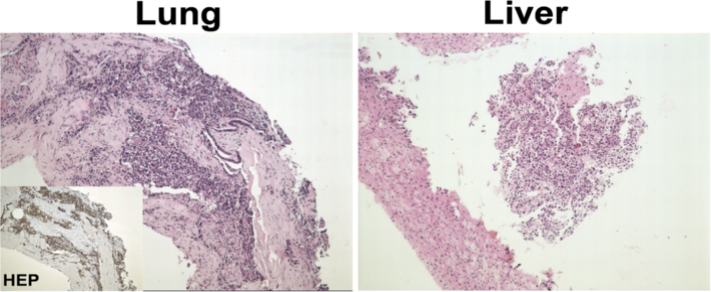
**Histopathological staining of fine needle aspiration biopsy for nodules in lung and liver (original magnification 100×).** The inset shows positive immunostaining for hepatocytes of the lung nodule biopsy. Immunostaining for both specimens indicated hepatocytes (+), α-fetoprotein (AFP) (+), thyroid transcription factor 1 (TTF-1) (−) and surfactant protein A (SPA) (−) (data not shown). HEP: hepatocyte.

A 36-year-old man with decompensate hepatitis-B-related cirrhosis underwent LDLT in our hospital on 23 June 2009. His younger brother, who was virus negative and had no evidence of tumor, donated the right lobe of his liver. The patient was hepatitis B surface antigen (HBsAg), hepatitis B E antigen (HBeAg) and hepatitis B core antibody (HBcAb) positive. The serum tests at the time of hospitalization showed that his HBV-DNA copy level was 2 × 10^8^/L accompanied by YMDD mutation (rtM204I). Because the patient had a 3-year history of lamivudine treatment, adefovir was then added on for treatment; 1 month later the serum HBV-DNA level decreased to 1 × 10^4^/L and he underwent LDLT. Preoperative ultrasonography, computed tomography and MRI all ruled out the presence of tumor. Histopathology of the explanted liver also showed no evidence of malignancy, but micronodular cirrhosis with lymphocytic infiltration in portal tracts and moderate steatosis in the cirrhotic nodules was seen. After LDLT, the patient received an immunosuppressive strategy consisting of tacrolimus, mycophenolate mofetil and prednisone. For antivirus treatment as well as prophylaxis of HBV recurrence, a combination of add-on adefovir plus lamivudine and hepatitis B immune globulin was used according to our protocol [12]. Serum HBsAg was continually negative and hepatitis B surface antibody (HBsAb) positive on multiple tests post transplantation until 1.5 years later, when HBsAg was detected and remained positive for the remainder of his follow-up sessions. At 2 years after LDLT, the patient complained of cough with fever. A thoracic computed tomography scan showed a lower left lung infection with a nodule in the hilum of the left lung; bronchofibroscopic biopsy of the same confirmed a bulk *Aspergillus* infection (Figure 1). After 2 weeks of antifungal treatment, however, his symptoms were not alleviated. Further ultrasonography and MRI showed a small nodule that was only 0.7 cm in diameter in segment VI of the allograft (Figure 2). At the same time, serum tests showed his α-fetoprotein (AFP) level was 400 ng/ml and a HBV-DNA copy test was positive. Fine needle aspiration biopsies were performed immediately for the nodule in the liver as well as a second time for the lung mass. Immunostaining for both specimens showed hepatocyte (+), AFP (+), thyroid transcription factor 1 (TTF-1) (−) and surfactant protein A (SPA) (−) indicating HCC (Figure 3). Thus, the diagnosis was HCC with lung metastasis with concurrent fungal infection. At the time of diagnosis, the patient had normal liver function. Therefore, his dose of tacrolimus was decreased by one-third and mycophenolate mofetil (MMF) maintained, but prednisone stopped. He underwent radiofrequency ablation for HCC in the liver and radiotherapy for the lung metastasis. At 3-month follow-up, his liver imaging screen was clear. However, more nodules were found in the lung, suggesting uncontrolled metastasis. Our patient died from respiratory failure due to rapid progression of the lung metastases 4 months later.

## Discussion

In the last few decades, increasing numbers of patients have been undergoing LTx worldwide. Owing to the maintenance therapy of immunosuppressive agents, the survival of transplant patients has greatly improved. However, an increased risk of *de novo* malignancies remains one of the major long-term complications after LTx. Potential factors related to *de novo* malignancies following LTx have been proposed as viral infection, long-term immunosuppressive therapy, longer survival of transplanted patients and possible malignant gene transfer from donors [13,14]. The reported predominant post-transplant *de novo* malignancies are post-transplantation lymphoproliferative disorder (PTLD) and skin tumors [15]. Other disorders such as gastric cancer, colorectal cancer, breast cancer and so on have also been reported [16,17]. Developing HCC after LTx most often happens in patients who have HCC before transplantation. Such cases are considered as HCC recurrence and have become a serious problem after LTx for HCC recipients. However, for non-tumor recipients, *de novo* occurrence of HCC is extremely rare, with only 11 cases reported in recent years [3-11]. In those cases, HCC developed from 3 to 22 years after the patient had received liver transplantation for end-stage liver diseases (HBV-related cirrhosis in four patients, HCV-related cirrhosis in four patients, alcoholic cirrhosis in two patients and Budd-Chiari syndrome in one patient). The present work reports a case of *de novo* HCC in the setting of HBV recurrence after LDLT for HBV-related cirrhosis.

Though the exact carcinogenic mechanisms are unknown, the etiology of HCC is strongly associated with cirrhosis of any cause, but is most related to chronic HBV or HCV infection [18]. Most of the reported *de novo* HCC cases were related to recurrent cirrhosis, either alcohol or viral related due to recurrent HBV or HCV infection [3,4,7,8]. Morita *et al*. reported a case of *de novo* HCC in a patient with sustained clearance of HCV after LDLT. Though cirrhosis had become established due to the HCV flare-up at 3 years after LDLT, successful clearance was obtained by treatments thereafter [11]. In the present case, HBV recurrence had also occurred prior to the diagnosis of *de novo* HCC. Faria *et al*. have suggested that pretransplant HBV-DNA viral load was positively associated with HBV recurrence at a median recurrence time of 15 months after transplantation despite receiving hepatitis B immunoglobulin, lamivudine and/or adefovir [19]. Our patient had a high load of HBV virus with YMDD mutation when he underwent LTx. Entecavir and tenofovir are newer antiviral drugs for HBV. Previous rescue therapy for chronic lamivudine-resistant HBV included switching to entecavir and adding adefovir or tenofovir. The lamivudine selected rtM204I/V mutant is part of the entecavir resistance profile and the tenofovir-lamivudine combination resistance profile [20]. At present, switching to entecavir is not recommended for rescue therapy of lamivudine-resistant HBV. Though individual data showed that tenofovir had good efficacy in blocking viral replication in HBV patients with lamivudine-resistant mutants [21], it has not been approved for HBV in China. A systemic review showed that lamivudine plus adefovir combined treatment was more effective and produced longer-lasting effects than other drugs to date [22]. Therefore, we applied adefovir add-on lamivudine antivirus treatment and the HBV-DNA copy level in our patient decreased dramatically. HBV was transiently cleared after transplantation and regular prophylaxis therapy was given, however, the patient still developed recurrent HBV infection 1.5 years after LDLT. Though the underlying mechanism was not clear, it is possible that the HBV recurrence could be attributed to the positive HBV-DNA viral load before transplant. This was also considered to be strongly associated with the HCC occurrence thereafter.

When considering *de novo* HCC, the time period from LTx to the diagnosis of *de novo* HCC is of interest. Reports in the literature show that most recurrent HCC occurred shortly after LTx, whereas *de novo* HCC usually develops several years after recurrent HBV or HCV infection; enough time to develop cirrhosis. According to the previously reported cases, the earliest cases of *de novo* HCC occurred 3 years after LTx, and the latest occurred 22 years after LTx. However, in the present case, *de novo* HCC developed only 2 years after LDLT/6 months after recurrent HBV. This was a rather short time for *de novo* HCC after LTx. Recently, a more precise genomic allelotyping method has been applied for distinguishing recurrent and *de novo* HCC after LTx. Multiple polymerase chain reaction amplification of the highly polymorphic short tandem report DNA sequences extracted from tumor tissues can effectively discriminate between donor and recipient origin [7,10,11]. This is extremely useful in cases where primary HCC existed before LTx [11,23]. However, some reports considered *de novo* HCC based on the clinical evidence rather than this method [5,6,8]. Use of this method was hampered in the present case due to technical reasons.

Neither pretransplant image studies nor intensive histopathology of the explanted liver revealed any evidence of malignancy existing before LTx. Moreover, evidence was clear that *de novo* HCC can develop in a short time after LTx. Vernadakis *et al*. reported a case of *de novo* HCC diagnosed 3 years after LTx without cirrhosis or any other clear reasons [10]. Indeed, HBV infection can lead to HCC in the absence of cirrhosis [18]. Therefore, it is not surprising that the recurrent HBV infection in the present case may be associated with the unusually rapid development of HCC without cirrhosis. However, viral YMDD mutation might also be associated with HCC development. Hosaka *et al*. reported that HCC could develop in HBV patients receiving adefovir add-on lamivudine treatment independently related to the YMDD mutation [24]. Thus, the positive HBV-DNA viral load and YMDD mutation before transplantation increased the risk of developing HBV recurrence and consequent elevated carcinogenesis after LTx under immunosuppression therapy in the present case. Taken together, though lacking direct genetic evidence of donor origin, we did observe HCC shortly after LTx that might be attributable to the HBV recurrence and YMDD mutation. To the best of our knowledge, this is the first report of *de novo* HCC in a liver graft with HBV recurrence after LTx for HBV-related liver cirrhosis with YMDD mutation.

Of interest, the present case of *de novo* HCC first presented as a lung mass followed by the detection of a minimal liver nodule that was only 7 mm. The patient only showed symptoms of pulmonary infection during the initial period. The existence of a pulmonary fungal infection obscured the primary diagnosis because fungal infection usually presents as a lung mass. We could not determine whether the fungal infection occurred before or as a result of obstruction of bronchi by the lung metastasis. Nevertheless, the fungal infection suggested a poor immunocompromised situation in the lung. Therefore, we presume that it might have contributed to the early lung metastasis despite a minimal liver involvement, thus compromising the therapeutic effect.

## Conclusions

*De novo* HCC is a rare finding in post-LTx patients and may be associated with recurrent HBV infection. Patients with positive HBV-DNA loads before LTx, especially those with pre-existing YMDD mutations, also have a higher risk of developing *de novo* HCC. In addition, unexplained fungal infection may also warrant evaluation as a possible cause of HCC development.

## Consent

Written informed consent was obtained from the patient’s next-of-kin for publication of this case report and any accompanying images. A copy of the written consent is available for review by the Editor-in-Chief of this journal.

## Abbreviations

AFP: α-Fetoprotein; HBsAg: Hepatitis B surface antigen; HBV: Hepatitis B virus; HCC: Hepatocellular carcinoma; HCV: Hepatitis C virus; LDLT: Living donor liver transplantation; LTx: Liver transplantation.

## Competing interests

The authors declare that they have no competing interests.

## Authors’ contributions

YS reviewed the data and literature and wrote the main manuscript. GH collected the patient data. ZL, YJ, YS and ZM were responsible for treatment of the patient. WW and ZS contributed to revising the manuscript. All authors have read and approved the final manuscript.
